# Proteome-wide genetic study identified therapeutic targets for early-onset and late-onset Alzheimer’s disease

**DOI:** 10.1097/JS9.0000000000005020

**Published:** 2026-03-04

**Authors:** Lin Chen, Hongxu Sun, Ming-Juan Fang, Nan Cheng, Yin Xu

**Affiliations:** Institute of Neurology, Anhui University of Chinese Medicine, Hefei, China

**Keywords:** drug target, early-onset Alzheimer’s disease, late-onset Alzheimer’s disease, protein biomarker, proteome-wide Mendelian randomization

## Abstract

**Backgrounds:**

Given the distinct pathogenic mechanisms of early-onset Alzheimer’s disease (EOAD) and late-onset Alzheimer’s disease (LOAD), identifying disease-specific therapeutic targets for each subtype is particularly critical.

**Methods:**

We performed proteome-wide Mendelian randomization (MR), colocalization analysis, summary-data-based MR, and Heterogeneity in Dependent Instruments (HEIDI) tests to identify the causal roles of candidate proteins in EOAD and LOAD. Further analyses included protein-protein interaction network analysis, GO/KEGG enrichment analyses, and single-cell RNA sequencing annotation. Druggability evaluation of the target proteins, Phenome-wide association study was conducted to systematically evaluate the potential adverse effects associated with druggable proteins. High-throughput molecular docking and molecular dynamics simulations were conducted to target the top therapeutic targets.

**Results:**

Genetically predicted levels of three proteins (APOE, NECTIN2, and PVR) were associated with EOAD risk, nine proteins (APOE, NECTIN2, PVR, EPHB4, SEMA3F, RNASET2, BTN1A1, PSAPL1, and GRN) were associated with LOAD risk, three proteins (APOE, NECTIN2, and PVR) were colocalized with EOAD, and six proteins (APOE, NECTIN2, PVR, SEMA3F, BTN1A1, and EPHB4) were colocalized with LOAD. Three of the proteins (APOE, NECTIN2, and PVR) serve as common targets for both EOAD and LOAD. EPHB4 and BTN1A1 were prioritized for LOAD with the most convincing evidence. The small molecule cucurbit[8]uril exhibits excellent binding affinity with both EPHB4 and BTN1A1 target proteins for the treatment of Alzheimer’s disease.

**Conclusions:**

This study pinpointed APOE, NECTIN2, and PVR as shared therapeutic targets for EOAD and LOAD, and EPHB4 and BTN1A1 singled out as a priority target for LOAD.

## Introduction

Alzheimer’s disease (AD), the leading cause of dementia, comprises 50–75% of all dementia diagnoses^[^1^]^. Aging is a well-established risk factor and key determinant for AD development^[^2^]^. The two main subtypes of the disease are early-onset Alzheimer’s disease (EOAD) and late-onset Alzheimer’s disease (LOAD), which are distinguished based on an arbitrary cutoff age at which symptoms typically begin to manifest, usually set at 65 years^[^3–5^]^. Among reported AD cases, EOAD constitutes 5–10%. Most cases of AD are LOAD. However, research on AD is predominantly of EOAD^[^6^]^. The mechanisms underlying these two types of AD and their associations with clinical, biomarker, and neuropathological changes remain unclear^[^7^]^. Therefore, there is a paucity of dedicated research specifically addressing drug treatments for EOAD and LOAD.

HIGHLIGHTS

- We performed proteome-wide Mendelian randomization (MR), colocalization analysis, summary-data-based MR, and HEIDI tests to identify the causal roles of candidate proteins in different types of Alzheimer’s disease.
- We conducted protein-protein interaction (PPI) analysis, GO/KEGG enrichment analyses, and single-cell RNA annotation to explore the biological functions of prioritized potential proteins.
- Our study identified APOE, NECTIN2, and PVR as shared targets for both EOAD and LOAD. We found the small molecule cucurbit[8]uril exhibits excellent binding affinity with both EPHB4 and BT1A1 target proteins, providing promising targets for the development of screening biomarkers and therapeutic agents for EOAD and LOAD.

Over the past several decades, substantial investments in research have been dedicated to uncovering disease-modifying interventions for AD. However, progress remains remarkably limited. Currently, most approved drugs included anti-amyloid protein drugs, cholinesterase inhibitors, glutamate regulator, yet there is a paucity of robust evidence to validate their substantial clinical benefits^[^8,9^]^. Notably, current anti-dementia therapeutics lack subtype-specific targeting for AD variants like EOAD or LOAD, instead offering primarily symptomatic management. The therapeutic stagnation in this field is largely attributed to the incomplete understanding of AD pathophysiology. Fortunately, genome-wide association studies (GWAS) in large human cohorts offer promising directions for developing innovative therapeutics for multifactorial disorders. Drug targets validated by genetic evidence show a markedly higher probability of success in preclinical and clinical development, as genetic signals enhance their biological plausibility and translational potential^[^10^]^. This study aimed to contribute to the characterization of plasma protein biomarkers in AD across a broader spectrum of biological pathways. However, only a fraction of the underlying genetic variation has been identified thus far, leaving the molecular mechanisms associated with different AD subtypes and their links to pathophysiological mechanisms, biomarkers, and drug targets largely unresolved.

Alongside GWAS, proteome-wide association studies have emerged as a powerful approach to elucidate the relationships between proteome abundance and phenotypic variations^[^11^]^, thereby bridging the gap between genetic architecture discovery and the elucidation of downstream protein mechanisms associated with human pathophysiology^[^12^]^. Integrative analysis frameworks that combine Mendelian randomization (MR) with colocalization methods have become indispensable tools for identifying biological mediators linking genetic factors to clinical outcomes^[^13^]^. MR leverages genetic variants as instrumental variables to estimate the causal effects of an exposure on an outcome, under the assumption that alleles are randomly allocated during meiosis, thereby minimizing confounding bias and reverse causation^[^14^]^. Statistical colocalization assesses whether two associated signals share common causal variants^[^15^]^. Although researchers have applied MR to identify the causal role of the brain protein abundance of seven genes (ACE, ICA1L, TOM1L2, SNX32, EPHX2, CTSH, and RTFDC1) in AD^[^16^]^, as well as druggable genes (EPHX2, SERPINB1, and SIGLEC11) for AD treatment^[^17^]^, no studies have specifically investigated age-related onset subtypes. Accordingly, we aimed to discover promising protein biomarkers and drug targets for EOAD and LOAD by integrating high-throughput blood proteomics with genetic data to determine the genomic architecture underlying protein levels in these subtypes.

In this study, we conducted a proteome-wide MR, colocalization analysis, summary-data-based Mendelian Randomization (SMR) and Heterogeneity in Dependent Instruments (HEIDI) test to identify therapeutic targets for EOAD and LOAD separately. First, a two-sample MR analysis was performed to estimate the causal effects of blood protein quantitative trait loci (pQTLs) on EOAD and LOAD. Second, colocalization analyses were carried out to verify the robustness of the expression instrumental variables. Third, SMR analysis was conducted to confirm the causal associations between gene expression and EOAD/LOAD, while the HEIDI test was applied to distinguish gene expression associated with EOAD/LOAD risk due to shared genetic variants rather than genetic linkage. Fourth, GO and KEGG analyses were performed to explore the potential mechanisms by which these proteins are involved in the pathogenesis of EOAD and LOAD. Fifth, single-cell RNA annotations were performed to further elucidate the molecular characteristics of these proteins. Phenome-wide MR was employed to assess the potential adverse effects of the identified proteins on EOAD/LOAD treatment. Then, the druggability of the target protein is evaluated. High-throughput molecular docking and molecular dynamics simulations were conducted to target the top therapeutic targets. Our study is compliant with the TITAN Guidelines 2025 – governing declaration and use of AI^[^18^]^.

## Materials and methods

### Study design

**Figure 1.**
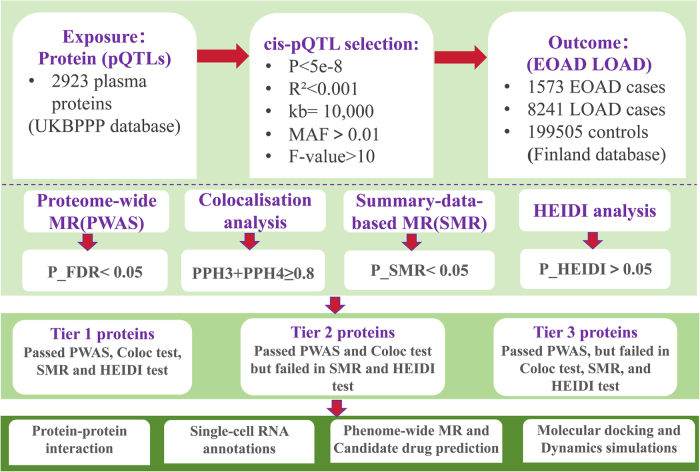
Flowchart of the study design. In this study, summary-level data on protein quantitative trait loci (pQTLs) and EOAD/LOAD were obtained from publicly accessible GWAS. Proteome-wide MR, colocalization analysis, SMR, HEIDI testing, PPI analysis, single-cell RNA annotations, and phenome-wide MR analyses were employed to identify potential protein biomarkers and therapeutic targets for EOAD and LOAD. EOAD, early-onset Alzheimer’s disease; HEIDI, heterogeneity in dependent instruments; LOAD, late-onset Alzheimer’s disease; MR, Mendelian randomization; PWAS, proteome wide association studies; SMR, summary-data-based Mendelian randomization.

As illustrated in Figure 1, our study employed a comprehensive approach encompassing proteome-wide MR, colocalization analysis, SMR, HEIDI testing, PPI analysis, single-cell RNA annotations, Phenome-wide MR, candidate drug prediction, molecular docking, and dynamics simulations. This multi-methodological strategy aimed to identify potential protein biomarkers and therapeutic targets for EOAD and LOAD. For this study, summary-level data on pQTLs and EOAD/LOAD were obtained from publicly accessible GWAS conducted among individuals of European ancestry. The data collection protocols were approved by the ethics committee overseeing the original GWAS, and all participants provided written informed consent before data acquisition, ensuring strict adherence to ethical standards.

### Study population and datasets

The current study included EOAD and LOAD cases and controls of European ancestry from the R12 version of the Finnish database (https://r12.finngen.fi/). A total of 1820 EOAD patients, 9690 LOAD patients, and 216 472 controls were included in the proteome-wide MR analysis. The diagnosis of EOAD and LOAD are determined based on the ICD-10 (International Classification of Diseases, 10th edition).

### Proteomic data source

The pQTLs were sourced from the UK Biobank-PPP database, representing the plasma proteomic profiles of 54 219 UK Biobank participants (https://pubmed.ncbi.nlm.nih.gov/37794186/). This represents one of the largest proteomic studies globally up to the present. In the proteome-wide MR research centered on drug targets, we selected pQTLs as instrumental variables, implementing specific screening criteria^[^19^]^. These criteria are outlined as follows: (1) The SNP within a vicinity of ±1 Mb around the gene region (cis-acting pQTLs); (2) A genome-wide significant threshold of *P* < 5 × 10^−8^ to identify highly correlated SNPs with plasma proteins; (3) To exclude linked SNPs and reduce linkage disequilibrium (LD) effects, an LD threshold of *r*^2^ < 0.001 and a genetic distance of 10 000 kb are applied; and (4) F-value greater than 10 to exclude weak instrumental variable bias.

### Proteome-wide MR analysis

We utilized the R package “TwoSampleMR” (V.0.6.0) for MR analysis to assess the associations between genetically predicted protein levels and EOAD/LOAD. For proteins with a single pQTL, the Wald ratio method was employed. For proteins with two or more pQTLs, we applied multiple approaches to estimate causality, including the inverse-variance-weighted (IVW) method, MR Egger, weighted median, and weighted mode-with IVW serving as the primary analytical approach^[^20,21^]^. Considering the multiple calculations involved, *P*-values from the MR analyses were adjusted for multiple testing using the Benjamini-Hochberg method to control the false discovery rate (FDR) at a threshold of 5%. Statistical significance was defined as an FDR value below 0.05^[^22,23^]^. Odds ratios (ORs) for the elevated risk of EOAD/LOAD were reported per standard deviation (SD) increase in plasma protein levels.

### Sensitivity analysis

We conducted a series of sensitivity analyses to verify the robustness of our findings. First, the Cochran Q test was employed to assess heterogeneity among genetic variants, with a P-value > 0.05 indicating no evidence of heterogeneity^[^21^]^. Second, the MR-Egger intercept was used to assess horizontal pleiotropy, where a *P*-value > 0.05 indicated the absence of horizontal pleiotropy^[^24,25^]^. Third, we utilized the MR Pleiotropy Residual Sum and Outlier (MR-PRESSO) test to identify instrumental variables that were influential outliers due to pleiotropy. A significant *P*-value (*P* < 0.05) was interpreted as evidence of potential horizontal pleiotropy, triggering the subsequent outlier correction step^[^26^]^. To bolster the robustness of the inferred causal relationship, we implemented Steiger filtering to validate the directional associations between the identified proteins and EOAD/LOAD. This method operates under the premise that a valid instrumental variable must account for a greater proportion of variance in the exposure (protein levels) than in the outcome (EOAD/LOAD)^[^27^]^. We leveraged the PhenoScanner GWAS database V2 (http://www.phenoscanner.medschl.cam.ac.uk/) and NHGRI-EBI GWAS catalog (https://www.ebi.ac.uk/gwas/home) to screen for single-nucleotide polymorphisms (SNPs) linked to potential confounders, including socioeconomic status, educational attainment, alcohol consumption, and smoking behavior^[^28–31^]^.

### Protein-protein interaction networks and enrichment analysis

Following the identification of numerous causally associated proteins through MR, we conducted a comprehensive Protein-Protein Interaction (PPI) analysis. This analysis aimed to explore both direct and indirect interactions among the identified proteins, while also validating their functional relevance in the pathogenesis of EOAD and LOAD. We utilized bioinformatics tools and databases, including STRING (https://string-db.org/) and Cytoscape, to systematically construct and analyze the PPI networks of EOAD- and LOAD-related proteins. We conducted functional and pathway enrichment analyses on the genes corresponding to the proteins identified via MR. We utilized the “clusterProfiler” and “enrichplot” R packages for enrichment analysis of Gene Ontology (GO) and Kyoto Encyclopedia of Genes and Genomes (KEGG) pathways. The GO analysis covered three main categories: Biological Processes (BP), Cellular Components (CC), and Molecular Functions (MF). A significance threshold of FDR < 0.05 was applied, and the top 10 most significant GO terms and KEGG pathways were visualized using the “ggplot2” R package.

### Bayesian colocalization analysis

Colocalization analysis was performed to confirm the presence of shared causal genetic variants between the exposure (protein levels) and outcome (EOAD/LOAD), thereby validating the MR findings. For proteins with significant MR results, we conducted colocalization analysis on SNPs within a ±1 MB window around the corresponding genes (cis-pQTLs) to assess their colocalization with EOAD/LOAD^[^32^]^. The posterior probability (PP) was used to quantify support for all the hypotheses, and they were identified as PPH0–PPH4: PPH0, no association with either trait; PPH1, association with the expression of the protein but not the EOAD/LOAD risk; PPH2, association with the EOAD/LOAD risk but not the protein; PPH3, association with the EOAD/LOAD risk and protein, with distinct causal variants; and PPH4, association with the EOAD/LOAD risk and protein, with a shared causal variant. A low PPH3 and PPH4 in combination with a high PPH0, PPH1 and/or PPH2 indicates limited power in the colocalization analysis^[^32,33^]^. Due to the limited statistical power in the colocalization analysis, we restricted our analysis to proteins with a combined PPH3 + PPH4 value of ≥0.8^[^17,34,35^]^.

### SMR and HEIDI analysis

We further performed SMR analysis^[^36^]^ as a complementary approach to validate the causal associations between proteins and EOAD/LOAD. The HEIDI test^[^37^]^ was employed, which uses multiple single-nucleotide polymorphisms (SNPs) within a genomic region to distinguish proteins associated with EOAD/LOAD risk due to shared genetic variants rather than genetic linkage. The SMR and HEIDI tests were conducted using the SMR software^[^38^]^. For the SMR analysis, the significance thresholds were set as follows: a *P*-value < 0.0167(0.05/3) for EOAD and a *P*-value < 0.0056 (0.05/9) for LOAD. A HEIDI test *P*-value > 0.05 indicated that the association between the protein and EOAD/LOAD was not driven by linkage disequilibrium.

### Single-cell RNA annotations

We obtained single-cell RNA-sequencing (scRNA-seq) data of brain tissue from the PanglaoDB database (https://panglaodb.se/), a user-focused single-cell sequencing resource developed for the scientific community. This database primarily features single-cell RNA sequencing datasets from both murine and human samples. We used the “Sample” module to search for two specific datasets, setting “Human” and “Tissue” as the screening criteria. The brain (neurons) database (SRA653146:SRS2874270) includes 8022 single-cell RNA-sequencing dataset samples. All publicly available datasets used in this study have obtained the required ethical approvals. Furthermore, the expression of the EPHB4 gene was investigated using the Human Protein Atlas (HPA) dataset (https://www.proteinatlas.org/). The HPA dataset comprises multiple sections, with this analysis primarily focusing on the tissue cell type panel and the single-cell type panel. By searching for the keyword “EPHB4” in the HPA dataset and selecting the “single-cell type” option, the expression levels of EPHB4 in brain cells can be retrieved. Selecting the “tissue cell type panel” provides a heatmap and bar chart illustrating the correlation between EPHB4 and marker genes of single cells within the cerebral cortex.

### Phenome-wide MR association study

To evaluate the potential adverse effects of protein targets, we performed Phenome-wide association study (PheWAS)^[^39^]^ analyses on the identified proteins using diseases as outcomes from the FinnGen cohort, which the outcome involved obtaining phenotypic data from the Finnish database in version R12 (encompassing 2469 phenotypes). In this study, the exposure was determined based on proteins that exhibited positive results in MR and colocalization methodology, and the criteria for selecting instrumental variables were consistent with those previously described. This extensive data set was used to perform PheWAS analysis, the causal effects are considered statistically significant at FDR < 0.05.

### Drug prediction, molecular docking and molecular dynamics simulation

We utilized five databases – namely DrugBank, Therapeutic Target Database, ChEMBL, and DGIdb – to predict existing drugs corresponding to five protein targets (APOE, NECTIN2, PVR, EPHB4 and BTN1A1) identified through analytical screening. High-throughput molecular docking was performed using a compound library of over 100 000 (see Supplemental Digital Content Table S13, available at: http://links.lww.com/JS9/H26). AlphaFold3 generated 3D structures of Tier 1 proteins (BTN1A1 and EPHB4)^[^40^]^. GHECOM identified their small-molecule binding pockets (max volumes: 1767 Å^3^ for BTN1A1, 32 524 Å^3^ for EPHB4), guiding box center coordinates and dimensions. Three rounds of screening were conducted with AutoDock Vina v1.2.5^[^41^]^: Round 1 (high-throughput, Exhaustiveness = 1, top 1% by affinity advanced); Round 2 (virtual screening, Exhaustiveness = 8, top 10% advanced); Round 3 (refined screening, Exhaustiveness = 64) for final affinity values. AutoDock Vina scoring criteria: High affinity (≤-10 kcal/mol); Good affinity (−7 to −10 kcal/mol). Molecular dynamics simulations were performed using GROMACS 2022. Force field parameters were generated via GROMACS’ pdb2gmx: CHARMM 36 for the receptor protein, and GAFF2 (Antechamber-generated, RESP charge assignment) for ligands. The system was solvated in a 1 nm cubic box with the TIP3P water mo GROMACS’ gmx genion added 0.15 M NaCl (Na⁺/Cl^−^) to ensure electroneutrality and physiological ionic strength. Long-range electrostatics were treated with the PME method (1 nm cutoff), force field/PME settings followed GROMACS specifications, and bond constraints used the LINCS algorithm. Prior to simulation, energy minimization was conducted (3000 steepest descent + 2000 conjugate gradient steps). Simulations employed the NPT ensemble (310 K via Nosé–Hoover thermostat, 1 bar via Parrinello–Rahman barostat) for 100 ns with a 2 fs integration step. GROMACS tools (g-rmsd, g-rmsf, g-hbond, g-Rg, and g-sasa) quantified RMSD, RMSF, HBonds, Rg, and SASA, respectively. MM-PBSA binding free energy of the complex was calculated using GROMACS’g_mmpbsa package.

## Results

### Proteome-wide MR analysis identified potentials of therapeutic targets for EOAD/LOAD

**Figure 2.**
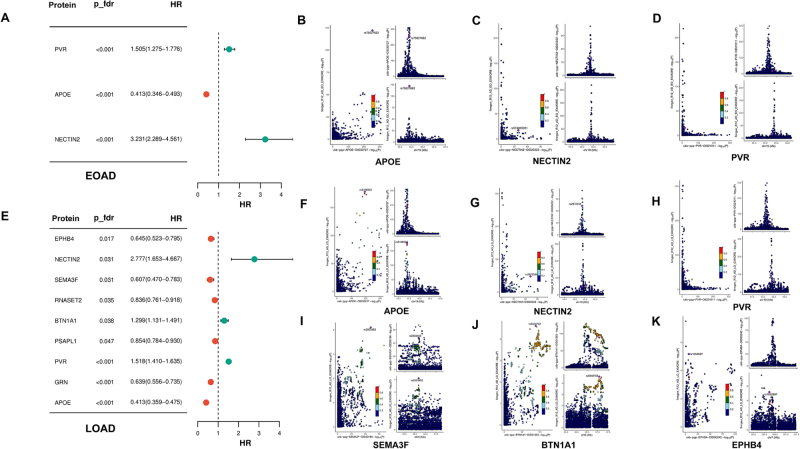
Forest plot of the proteome-wide MR and Colocalization results. (A) Forest plot of EOAD-associated proteins (*FDR < 0.05*). (B–D) Colocalization result of EOAD-associated proteins. (E) Forest plot of LOAD-associated proteins (*FDR < 0.05*). (F–K) Colocalization result of LOAD-associated proteins. MR, Mendelian randomization; EOAD, early-onset Alzheimer’s disease; LOAD, late-onset Alzheimer’s disease; FDR, False Discovery Rate .

All genetic instruments exhibited F-statistics exceeding 10, demonstrating strong statistical power and reliability. The instrumental variables ultimately used for the Proteome-wide MR analysis are presented in the Supplemental Digital Content Table S1, available at: http://links.lww.com/JS9/H14. A *P*-value of IVW or Wald ratio less than 0.05 is considered to have a suggestive association. A total of 90 proteins exhibited suggestive associations with EOAD risk (*P* < 0.05), and 141 proteins exhibited suggestive associations with LOAD risk (*P* < 0.05) (Supplemental Digital Content Table S2, available at: http://links.lww.com/JS9/H15). After FDR correction, genetically predicted levels of three proteins (APOE, NECTIN2, and PVR) were significantly associated with EOAD risk (FDR < 0.05) (Fig. 2A), nine proteins (APOE, NECTIN2, PVR, EPHB4, SEMA3F, RNASET2, BTN1A1, PSAPL1, and GRN) were significantly associated with LOAD risk (FDR < 0.05; Fig. 2E) (Supplemental Digital Content Table S3, available at: http://links.lww.com/JS9/H16). Genetically predicted higher levels of NECTIN2 (*P* = 2.57E^−11^, OR = 3.23) and PVR (*P* = 1.38E^−07^, OR = 1.50) were associated with an increased risk of EOAD, while the higher levels of the APOE (*P* = 7.96E^−23^, OR = 0.41) was associated with a lower risk of EOAD. Genetically predicted higher levels of NECTIN2 (*P* = 1.15E^−04^, OR = 2.78), PVR (*P* = 2.13E^−28^, OR = 1.52) and BTN1A1 (*P* = 2.11E^−4^, OR = 1.30) were associated with an increased risk of LOAD, while higher levels of APOE (*P* = 2.31E^−35^, OR = 0.42), EPHB4 (*P* = 3.93E^−05^, OR = 0.64), GRN (*P* = 3.43E^−10^, OR = 0.64), SEMA3F (*P* = 1.24E^−4^, OR = 0.61), PSAPL1(*P* = 2.95E^−4^, OR = 0.85) and RNASET2 (*P* = 1.65E^−04^, OR = 0.84) were associated with a lower risk of LOAD. These associations were generally consistent in additional analyses, including weighted mode, weighted median, and MR-Egger. No heterogeneity and pleiotropy were found (*P_heterogeneity_* > 0.05, *P_pleiotropy_* > 0.05) (Supplemental Digital Content Table S4, available at: http://links.lww.com/JS9/H17, Supplemental Digital Content Table S5, available at: http://links.lww.com/JS9/H18). All results of the discovery proteome-wide MR are shown in Supplemental Digital Content Table S6, available at: http://links.lww.com/JS9/H19.

### PPI network and enrichment analysis of the potentials of therapeutic targets

**Figure 3.**
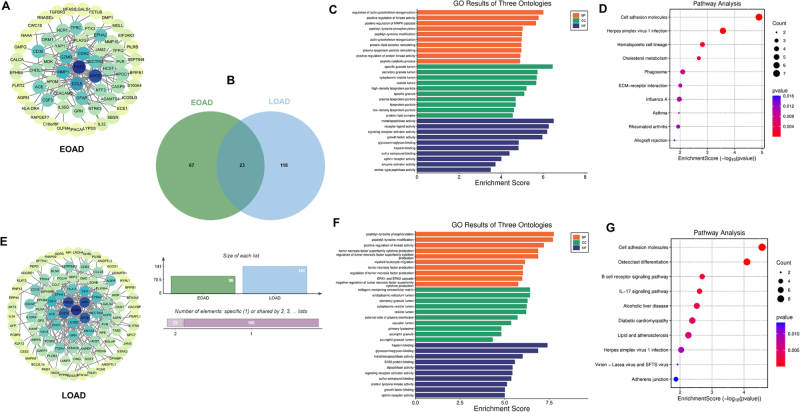
PPI Network and enrichment analysis of EOAD- and LOAD-associated proteins. (A) PPI network diagram of EOAD-associated proteins. (B) Venn diagram of EOAD-associated proteins and LOAD-associated proteins. (C) The top 10 terms in BP, CC, and MF of GO enrichment are presented in the bar plot of EOAD. (D) The top 10 terms in KEGG are depicted in the bubble plot of EOAD. (E) PPI network diagram of LOAD-associated proteins. (F) The top 10 terms in BP, CC, and MF of GO enrichment are presented in the bar plot of LOAD. (G) The top 10 terms in KEGG are depicted in the bubble plot of LOAD. EOAD, early-onset Alzheimer’s disease; LOAD, late-onset Alzheimer’s disease; PPI, protein–protein interaction.

We utilized Cytoscape to construct and visualize protein – protein interaction (PPI) networks for EOAD-associated proteins (Fig. 3A) and LOAD-associated proteins (Fig. 3E). Venn diagrams revealed 23 identified overlapping proteins between EOAD and LOAD (Fig. 3B). GO and KEGG enrichment analyses were performed using 90 and 141 genetically predicted proteins for EOAD and LOAD, respectively, identified via MR. For EOAD, we obtained 241 statistically significant (*FDR < 0.05*) GO function enrichment entries, including a total of 171 biological process (BP), 38 molecular function (MF), and 32 cellular component (CC) terms (Supplemental Digital Content Table S7, available at: http://links.lww.com/JS9/H20). The top 10 terms in BP, CC, and MF were selected and plotted in the column chart (Fig. 3C). The KEGG pathways included cell adhesion molecules, phagosome, and PI3K-Akt signaling pathway (Fig. 3D; Supplemental Digital Content Table S7, available at: http://links.lww.com/JS9/H20). For LOAD, we obtained 436 statistically significant (*FDR < 0.05*) GO function enrichment entries, including a total of 358 BP, 52 MF, and 26 CC terms (Supplemental Digital Content Table S8, available at: http://links.lww.com/JS9/H21). The top 10 terms in BP, CC, and MF were selected and plotted in the column chart (Fig. 3F). The KEGG pathways included cell adhesion molecules, Toll-like receptor signaling pathway, HIF-1 signaling pathway (Supplemental Digital Content Table S8, available at: http://links.lww.com/JS9/H21). The top 10 potential signaling pathways were presented as bubble plots (Fig. 3G).

### Colocalization analysis supported the causality of 3 EOAD-associated proteins and 6 LOAD-associated proteins

We performed co-localization analysis on the three EOAD-related proteins and nine LOAD- related proteins that were significantly associated as identified through proteome-wide MR. Among them, three EOAD-related proteins (APOE, NECTIN2, and PVR) showed strong genetic colocalization evidence (PPH3 + PPH4 > 0.8) (Fig. 2B–D, Table 1). Additionally, six LOAD-related proteins (APOE, NECTIN2, PVR, SEMA3F, BTN1A1, and EPHB4) also exhibited strong genetic colocalization evidence (PPH3 + PPH4 > 0.8; Fig. 2F–K, Table 1). This suggests a high probability of shared causal variants between protein and the risk of EOAD or LOAD. Notably, three proteins are common targets for both EOAD and LOAD. These proteins are APOE, NECTIN2, and PVR. There was weak evidence of colocalization between RNASET2, PSAPL1, GRN and LOAD. The colocalization results are provided in Table 1 and Supplemental Digital Content Table S9, available at: http://links.lww.com/JS9/H22.

**Table 1:** Results of proteome-wide MR, colocalization, SMR, and HEIDI test for EOAD-related proteins and LOAD-related proteins.

Proteome-wide MR				Colocalization	SMR	HEIDI analysis	
Exposure	Outcome	Method	p_FDR	H3 + H4	p_SMR	p_HEIDI	Targets
APOE	EOAD	IVW	1.01E−19	1.00	1.95E−198	3.89E−28	Tier 2
NECTIN2	EOAD	IVW	1.63E−08	1.00	4.40E−05	1.56E−13	Tier 2
PVR	EOAD	IVW	5.85E−04	1.00	2.23E−01	6.72E−04	Tier 2
APOE	LOAD	IVW	2.93E−32	1.00	0	0	Tier 2
PVR	LOAD	IVW	1.35E−25	1.00	8.09E−05	6.68E−09	Tier 2
RNASET2	LOAD	IVW	4.50E−02	0.55	6.21E−03	8.30E−01	Tier 3
EPHB4	LOAD	IVW	1.66E−02	0.99	1.29E−03	1.64E−01	Tier 1
GRN	LOAD	Wald ratio	2.17E−07	0.99	7.13E−10	2.05E−04	Tier 2
SEMA3F	LOAD	IVW	3.14E−02	0.81	1.86E−01	9.97E−01	Tier 2
BTN1A1	LOAD	IVW	3.82E−02	0.93	2.55E−04	9.21E−01	Tier 1
NECTIN2	LOAD	IVW	3.14E−02	1.00	3.62E−13	2.25E−36	Tier 2
PSAPL1	LOAD	IVW	4.68E−02	0.69	2.84E−02	8.28E−01	Tier 3

EOAD, early-onset Alzheimer’s disease; FDR, False Discovery Rate; HEIDI, Heterogeneity in Dependent Instruments; LOAD, late-onset Alzheimer’s disease; MR, Mendelian randomization; SMR, summary-data-based Mendelian randomization.

### SMR and HEIDI tests verified one causal protein

To further validate the observed findings, we conducted SMR and HEIDI tests for nine proteins with complete summary-level data. Among these, two proteins passed the SMR test for EOAD (*P* < 0.0125), and six proteins passed the SMR test for LOAD (*P* < 0.0056; Table 1). Five proteins passed the HEIDI test for LOAD (*P* > 0.05), whereas no protein passed the HEIDI test for EOAD (*P* > 0.05; Table 1). The results of SMR and HEIDI test are presented in Supplemental Digital Content Table S10, available at: http://links.lww.com/JS9/H23. Based on the aforementioned evidence, we categorized these proteins into three tiers. Two proteins (EPHB4, BTN1A1) passed all tests and were classified into Tier 1. Five proteins (APOE, NECTIN2, PVR, GRN, and SEMA3F) passed the colocalization analysis but failed the SMR and HEIDI test and were classified into Tier 2. Two proteins (RNASET2 and PSAPL1) failed both the colocalization analysis and the SMR and HEIDI test and were classified into Tier 3 (Table 1).

### Single-cell RNA annotations

**Figure 4.**
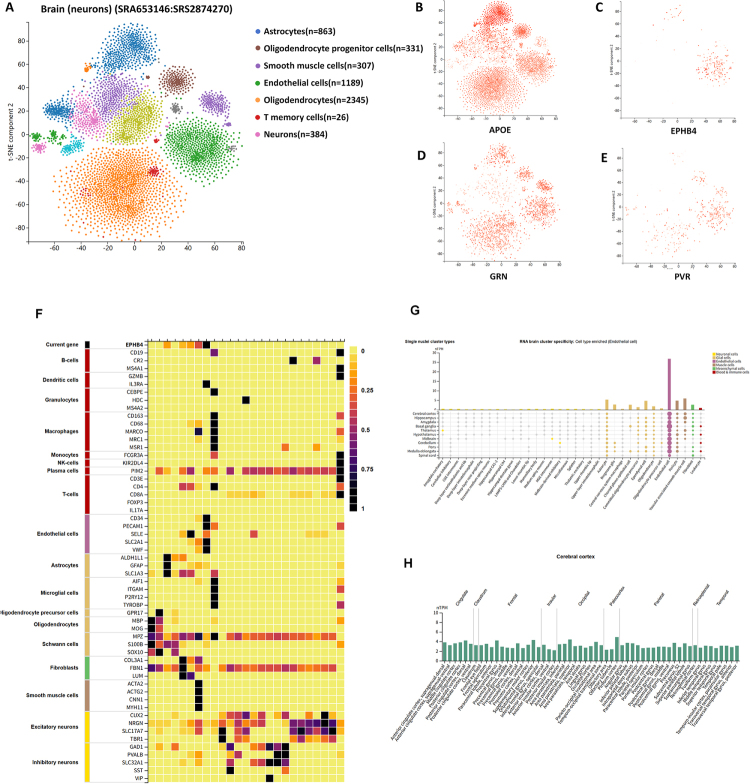
Single-cell RNA annotations. (A) Single – cell RNA annotation dataset for brain (neurons) (SRA653146:SRS2874270). (B) Expression of the APOE gene. (C) Expression of the *EPHB4* gene. (D) Expression of the *GRN* gene. (E) Expression of the *PVR* gene. (F) The heatmap illustrates the correlation analysis of the *EPHB4* gene with other genes in single cells of the cerebral cortex. (G) RNA brain cluster specificity of the *EPHB4* gene. (H) Expression of the *EPHB4* gene in the different Cerebral Cortex.

We selected one data set brain (neurons; SRA653146:SRS2874270; Fig. 4A) and analyzed the expression levels of seven proteins corresponding to the genes (EPHB4, BTN1A1, APOE, NECTIN2, PVR, GRN, and SEMA3F) in the dataset. The single-cell RNA annotations for brain (neurons) cluster specificity revealed that the *APOE* gene and *GRN* gene exhibited highly expressed in all brain cells types (astrocytes, oligodendrocyte progenitor cells, smooth muscle cells, endothelial cells, oligodendrocytes, T memory cells, and neurons; (Fig. 4B and D). *EPHB4* gene was highly expressed in astrocytes cells and endothelial cells (Fig. 4C). *PVR* gene was highly expressed in endothelial cells and oligodendrocytes (Fig. 4E). The other genes, such as *NECTIN2, RNASET2*, and *BPIFB1*, exhibit no expression at all. Upon querying the Human Protein Atlas (HPA) database, it was revealed that the RNA brain cluster specificity of the EPHB4 gene exhibits cell-type enrichment, particularly in endothelial cells, and is also associated with astrocytes, ependymal cells, vascular-associated smooth muscle cells, and pericytes (Fig. 4G and F). The EPHB4 gene exhibits high expression in the different cerebral cortex, specifically within the piriform cortex, parastriate area, superior and anterior orbitofrontal gyri, posterior precentral gyrus, dorsal posterior cingulate cortex, and dorsal posterior cingulate cortex (Fig. 4H).

### Phenome-wide MR analysis of EOAD and LOAD prior protein targets

**Figure 5.**
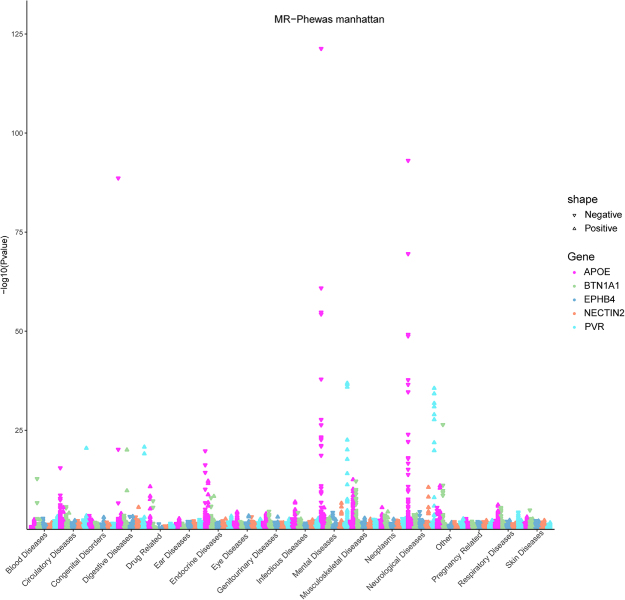
Manhattan plot for phenome-wide MR results of EPHB4, APOE, NECTIN2, PVR, and BTN1A1. Ordinate representation of the *P*-value in phenome-wide MR results. A dot represents a disease trait, and different colors represent the MR result of different expressions.

We evaluated the side effects of Tier 1 proteins (EPHB4 and BTN1A1) and those of Tier 2 proteins (APOE, NECTIN2, and PVR) that are common targets for both EOAD and LOAD through a comprehensive PheWAS analysis, screening 2469 phenotypes from the Finnish database (version R12). Overall, we identified significant causal associations between BTN1A1, EPHB4, APOE, NECTIN2, PVR, and 152 phenotypes (*FDR < 0.05*; Fig. 5; Supplemental Digital Content Table S11, available at: http://links.lww.com/JS9/H24). APOE may be associated with an increased risk of several adverse conditions, including gonarthrosis, type 2 diabetes, polyarthropathies, hypertensive heart disease, and severe cardiovascular diseases (*OR > 1, FDR < 0.05*). BTN1A1 may be linked to sarcoidosis, autoimmune hyperthyroidism, osteomyelitis, inflammatory bowel disease, arthrosis, and cardiovascular diseases (*OR < 1, FDR < 0.05*). EPHB4 may be implicated in endometriosis of the ovary, anterior iridocyclitis, acute and subacute iridocyclitis, ulcerative ileocolitis, and iridocyclitis (*OR > 1, FDR < 0.05*). No significant associations were observed between NECTIN2 or PVR and any of the investigated diseases after correction for multiple testing (*FDR: 0.05*). Summary results are provided in Supplemental Digital Content Table S12, available at: http://links.lww.com/JS9/H25.

### Druggability evaluation of the target proteins

The results indicated that five proteins (APOE, NECTIN2, PVR, EPHB4, and BTN1A1) have been identified as potential targets for drug development. APOE is druggable, making it a promising target for the development of AD. CN-105 is a well-established APOE-mimetic peptide that exerts neuroprotective effects by promoting amyloid-beta clearance and exhibiting anti-inflammatory properties. Its amino acid sequence is Acetyl-Valine-Serine-Arginine-Arginine-Arginine-NH2 (Ac-VSRRR-NH2). Regarding the PVR protein, its targeted drug Lerapolturev is currently in Phase II clinical trials, with indications covering glioblastoma multiforme, refractory melanoma, and recurrent glioblastoma multiforme. For the EPHB4 protein, the research and development progress of its targeted drug (sEphB4-HSA) varies by formulation: some iterations are in Phase II/III clinical trials for the treatment of metastatic urothelial carcinoma, muscle-invasive bladder cancer, and Kaposi’s sarcoma; meanwhile, sEphB4-HAS developed as an antagonist is in Phase II clinical trials, with transitional cell carcinoma as its indicated disease. Phloretin is a small molecule associated with the BTN1A1 protein and demonstrates anti-inflammatory and antioxidant effects. To date, no targeted drugs against the NECTIN2 protein have been approved for marketing or entered the clinical stage. Although four protein targets are considered druggable, no drugs targeting PVR, BTN1A1, or EPHB4 have been approved for the treatment of AD. These findings suggest significant potential for developing new therapeutic strategies targeting these proteins for the treatment of AD.

### Molecular docking and molecular dynamics simulations

**Figure 6.**
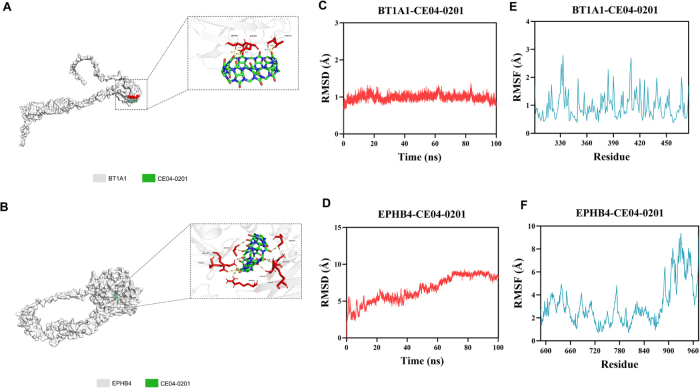
Molecular docking models and molecular dynamics simulations of protein-ligand complexes. (A) Macroscopic 3D molecular docking model of BTN1A1-CE04-0201. (B) Macroscopic 3D molecular docking model of EPHB4-CE04-0201. (C) Variations in RMSD of the BTN1A1-CE04-0201 complex during molecular dynamics simulations. (D) Variations in RMSD of the EPHB4-CE04-0201 complex during molecular dynamics simulations. (E) Variations in RMSF of the BTN1A1-CE04-0201 complex during molecular dynamics simulations. (F) Variations in RMSF of the EPHB4-CE04-0201 complex during molecular dynamics simulations.

Tier 1 (EPHB4 and BTN1A1) proteins and compounds were screened through three high-throughput molecular docking. The results showed that the compound with the highest binding affinity to both EPHB4 and BTN1A1 was the same: CE04-0201 (Cucurbit[8]uril) (https://www.chemdiv.com/catalog/inhibitors/compound-CE04-0201/). The maximum affinity between BTN1A1 and CE04-0201 was −11.18 kcal/mol (Fig. 6A), and all the docking results of BTN1A1 with compounds are presented in Supplemental Digital Content Table S14, available at: http://links.lww.com/JS9/H27. The maximum affinity between EPHB4 and CE04-0201 was −14.07 kcal/mol (Fig. 6B), and all the docking results of EPHB4 with compounds are presented in Supplemental Digital Content Table S15, available at: http://links.lww.com/JS9/H28. Both sets of protein-compound docking were considered to be high-affinity binding. The BTN1A1-CE04-0201 complex system reached equilibrium after 20 ns and subsequently exhibited stable fluctuations around 1.1 Å (Fig. 6C). In contrast, the EPHB4-CE04-0201 complex system displayed a gradual increase in RMSD with relatively moderate fluctuations, achieving equilibrium after 70 ns and remaining below 9 Å throughout the simulation (Fig. 6D). The BTN1A1-CE04-0201 complex exhibited relatively low RMSF values (mostly below 2.2 Å), indicating low flexibility and high stability (Fig. 6E). The EPHB4-CE04-0201 complex had RMSF values mostly below 9 Å, thus showing high structural flexibility (Fig. 6F). Both the EPHB4-CE04-0201 and BTN1A1-CE04-0201 complex systems showed stable binding with well-formed hydrogen bonding interactions. The time-dependent Rg values, SASA values, HBonds values, and binding free energy of the protein-ligand complex calculated are shown in Supplemental Digital Content Figure 1A–I, available at: http://links.lww.com/JS9/H13. Therefore, the small molecule CE04-0201 exhibits favorable binding affinity with both EPHB4 and BTN1A1 target proteins.

## Discussion

The discovery of innovative agents for EOAD/LOAD remains a formidable challenge. This hurdle primarily stems from the incomplete understanding of EOAD/LOAD pathophysiology. In this comprehensive study, we explored the causal relationships between a large panel of plasma proteins and the risk of EOAD/LOAD. Our findings revealed nine proteins that may modulate EOAD/LOAD disease outcomes. Three proteins (APOE, NECTIN2, and PVR) serve as common targets for both EOAD and LOAD. Bayesian colocalization highlighted the causal effects of six protein biomarkers for EOAD and LOAD, and two proteins were verified by SMR and HEIDI tests. Collectively, we identified two proteins (EPHB4 and BTN1A1) with the most convincing evidence (tier 1), five proteins (APOE, NECTIN2, PVR, GRN, and SEMA3F) with convincing evidence (tier 2), and two proteins (RNASET2 and PSAPL1) with middle convincing evidence (tier 3). The EPHB4 gene was highly expressed in astrocytes cells and endothelial cells of brain. Moreover, Phenome-wide MR analysis demonstrated that treatments targeting EPHB4 and BTN1A1 had a few potential side effects. The small molecule cucurbit[8]uril exhibits excellent binding affinity with both EPHB4 and BTN1A1 target proteins for the treatment of AD.

Developing drugs requires a detailed understanding of how a particular gene or genetic variant contributes to the disease process^[^42^]^. Without a clear understanding of the molecular mechanisms, it is challenging to design drugs that specifically target the relevant pathways or processes. Over the past decade, the widespread application of GWAS has revolutionized the field AD research. By simultaneously analyzing hundreds of thousands to millions of genetic markers across the entire human genome in large cohorts of AD patients and healthy controls, GWAS have successfully identified over 100 genetic loci significantly associated with AD risk^[^43,44^]^. These discoveries include not only well – known genes such as APOE, which has long been recognized as a major genetic determinant of AD, but also numerous novel genes involved in diverse biological processes^[^45^]^. While GWASs have made significant progress in identifying AD-associated genetic variants, there is still a long way to go in understanding the underlying mechanisms and translating these findings into effective therapeutic strategies^[^46^]^. Different AD subtypes have different pathogenesis and clinical outcomes. Therefore, we are actively seeking their respective molecular targets. Our study identified 92 proteins associated with EOAD and 123 proteins associated with LOAD. Among these, 24 overlapping proteins were screened as potential common biomarkers for both EOAD and LOAD. EOAD and LOAD share the same KEGG pathways: cell adhesion molecules. EOAD is also associated with cytokine-cytokine receptor interactions and apoptosis. LOAD, on the other hand, is related to the lysosome, B-cell receptor signaling pathway. After multiple corrections and co-localization analyses, APOE, NECTIN2, and PVR were identified as common targets for both EOAD and LOAD. Additionally, EPHB4 and BTN1A1 were prioritized as targets specifically for LOAD. This finding has not been previously reported in the literature.

EPHB4 emerges as a novel, high-confidence candidate from our analysis. While it has not been extensively studied in the context of AD, with only sporadic reports^[^47^]^, its well-established biological functions align closely with multiple core pathophysiological pathways of AD. EPHB4 is a critical regulator of angiogenesis and vascular integrity^[^48,49^]^, and play important roles in neovascularization and arteriovenous differentiation/plasticity^[^50^]^. Given the growing recognition of cerebrovascular dysfunction and blood-brain barrier breakdown in AD pathogenesis, it is plausible that genetic variations in EPHB4 influence AD risk by compromising cerebral vasculature, thereby impairing Aβ clearance and promoting neuroinflammation. More compellingly, EPHB4 plays a key role in synaptic plasticity^[^51^]^, which are fundamental to learning and memory. Intriguingly, the closely related family member EPHB2 has been identified as a synaptic target for Aβ oligomers^[^52^]^, whose binding leads to EphB2 degradation and subsequent synaptic failure. We hypothesize that EPHB4 may represent a parallel or compensatory pathway within the same receptor family that confers resistance to synaptic toxicity in AD. Therefore, the robust genetic evidence for EPHB4 from our study, coupled with its plausible roles in vascular and synaptic mechanisms, positions it as a previously underappreciated therapeutic target for AD. Future studies are warranted to elucidate its precise cell-type-specific functions in AD models.

NECTIN2, also known as PVRL2, is a cell adhesion molecule extensively studied in immune regulation and virology, but also expressed in the brain where it facilitates neuron-glia and glia-glia interactions^[^53,54^]^. The NECTIN2 gene demonstrated a highly significant correlation with the risk of AD in observational studies^[^55,56^]^. As a ligand for the immune receptor TIGIT on T and NK cells, the NECTIN2-TIGIT axis is a known checkpoint for suppressing immune responses^[^57^]^. Recent study indicates that neuroinflammation is associated with AD and its progression. This process involves both the activation of resident immune cells in the brain and the infiltration of activated immune cells from peripheral sources^[^58^]^. We hypothesize that dysregulation of NECTIN2 in the AD brain could modulate neuroinflammation by altering the activity of infiltrating or resident immune cells, thereby influencing disease progression. Recent research suggests that alterations in the connectivity and dysfunction of the blood-brain barrier (BBB) may play a significant role in the onset of AD. The integrity of the BBB is maintained by various connections, including adherence junctions which involve molecules such as endothelial NECTIN^[^59^]^. As an adhesion molecule, NECTIN2 contributes to the formation of adherens junctions^[^60^]^. Disruptions in these junctions or their components can lead to increased permeability and impair neuronal signaling, both of which are closely linked to the progression of AD. The disturbance to autophagy-lysosomal pathway can cause proteins to accumulate, resulting in pathological process of AD^[^61^]^. Nectin2 is ubiquitinated on tumor cells and that this modification can promote protein degradation^[^62^]^. However, there are currently no reports on NECTIN2 studies in lysosomal systems of AD. We conclude that our genetic evidence, positioning NECTIN2 as a causal risk factor, is highly consistent with its dual potential role in modulating both neuroinflammation and cellular barrier functions, providing a compelling mechanistic hypothesis for future functional studies.

BTN1A1 is a member of the immunoglobulin superfamily, and was initially discovered to be involved in the immune regulation of the central nervous system within the context of butyrophilin family functions. Butyrophilins are known to play a crucial role in the immune response and cell adhesion^[^63^]^. As an immunomodulatory protein, BTN1A1 may influence neuroinflammation^[^64^]^, a core component of the pathogenesis of AD. We hypothesize that dysregulation of BTN1A1 may alter the activity of microglia, the brain’s resident immune cells, potentially leading to the chronic neuroinflammatory state in AD. Given that BTN1A1 is structurally associated with the immunoglobulin superfamily, it may play a role in cell adhesion between neurons and glial cells^[^65^]^. Impairment of this adhesion function could impair synaptic stability and the integrity of neuronal networks. We conclude that our robust genetic evidence suggests that BTN1A1 is a pathogenic risk factor, which is highly consistent with its potential dual role in regulating neuroinflammation and cell adhesion.

In our study, the small molecule Cucurbit[8]uril showed good binding affinity to both EPHB4 and BTN1A1 target proteins. Cucurbit[8]uril is not merely a novel compound but a versatile supramolecular platform with great potential applications in the biomedical field^[^66,67^]^. The central cavity of Cucurbit[8]uril is well-suited for encapsulating key aromatic residues that are crucial for the self-assembly and aggregation of Aβ peptides^[^66^]^. By isolating these residues, Cucurbit[8]uril is expected to inhibit the formation of toxic Aβ oligomers and fibrils, which are central to AD pathology. Similarly, Cucurbit[8]uril could interact with critical residues in the tau protein, mitigating its hyperphosphorylation and aggregation into neurofibrillary tangles. Many potential neuroprotective drugs suffer from poor water solubility and low bioavailability. Cucurbit[8]uril can form inclusion complexes with these drugs, significantly improving their solubility and protecting them from degradation, thereby enhancing their therapeutic index^[^67^]^. Cucurbit[8]uril affinity can be used in biosensors to detect low concentrations of AD biomarkers or drugs in cerebrospinal fluid or blood, thereby enabling more accurate diagnosis and monitoring^[^68^]^. So, Cucurbit[8]uril represents a paradigm shift in AD treatment – from traditional receptor inhibition to supramolecular intervention at the protein aggregation level and targeted drug delivery.

There are several limitations to this study that warrant consideration. First, the inclusion of only participants of European descent may restrict the generalizability of the results to other populations. For instance, the effect sizes and allele frequencies of well-established risk genes like APOE are known to differ across populations, and it is plausible that the causal roles of the proteins we identified may also exhibit ancestry-specific heterogeneity. Therefore, our results should be interpreted as preliminary evidence primarily applicable to European populations. A critical future direction is the replication of these analyses in large-scale, multi-ancestry cohorts to ensure equitable translation of genetic findings. Additionally, while this study utilized multiple methods to validate the reliability of the results, the absence of accessible data in other databases regarding EOAD and LOAD prevented the inclusion of multi-database joint verification, which might have further strengthened the findings. This limitation prompted us to analyze the underlying causes, avoid exaggeration, and emphasize the necessity of future replication experiments on larger, phenotypic datasets. We look forward to further investigating these drug targets and their potential therapeutic implications for EOAD/LOAD in future studies.

## Conclusion

Our study identified APOE, NECTIN2, and PVR as shared targets for both EOAD and LOAD, with EPHB4 and BTN1A1 prioritized specifically for LOAD. Collectively, our findings provide genetic evidence supporting the therapeutic potential of EPHB4 and BTN1A1 for LOAD, which may inform prioritization strategies in LOAD drug development pipelines. The small molecule cucurbit[8]uril exhibits excellent binding affinity with both EPHB4 and BTN1A1 target proteins for the treatment of AD.

## Data Availability

The original contributions presented in the study are included in the article/Supplementary Material. Further inquiries can be directed to the corresponding authors.

## Research registration unique identifying number (UIN)

Not applicable. The data collection protocols were approved by the ethics committee overseeing the original GWAS. We are grateful to the UK Biobank and the Finnish database for supplying data on summary statistics for MR analyses. We also want to thank all the researchers who shared these data and the study participants.
